# Supplementary figures and images for: A total of 219 metagenome-assembled genomes of microorganisms from Icelandic marine waters
Source: PeerJ. 2021 Apr 2;9:e11112. doi: 10.7717/peerj.11112 (PMC8020865; doi:10.7717/peerj.11112)

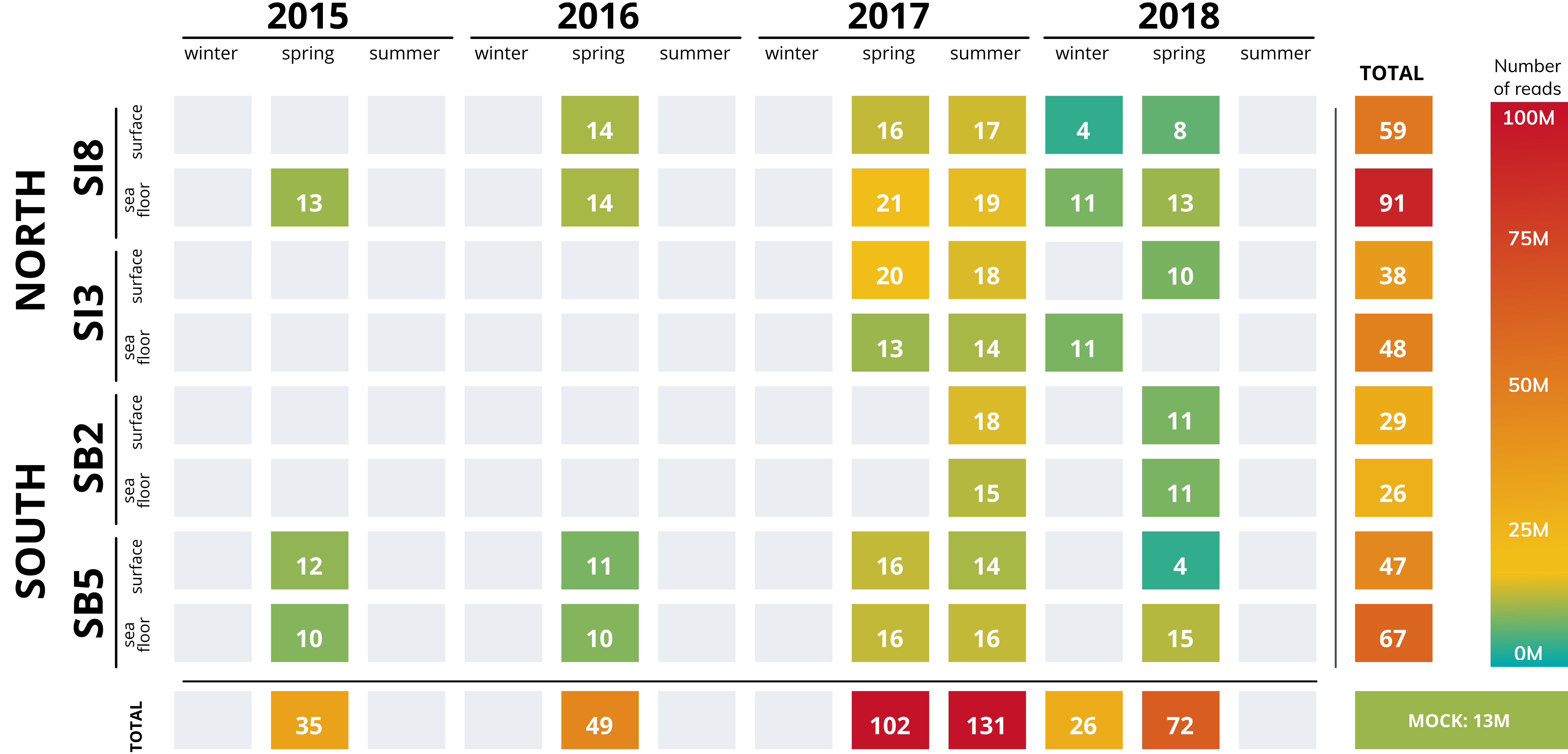

Supplement: Supplemental Information 1 — Metagenomic datasets from 32 samples (31 seawater samples and mock community). Number of reads displayed depending on the sampling locations and times. [file peerj-09-11112-s001.png]

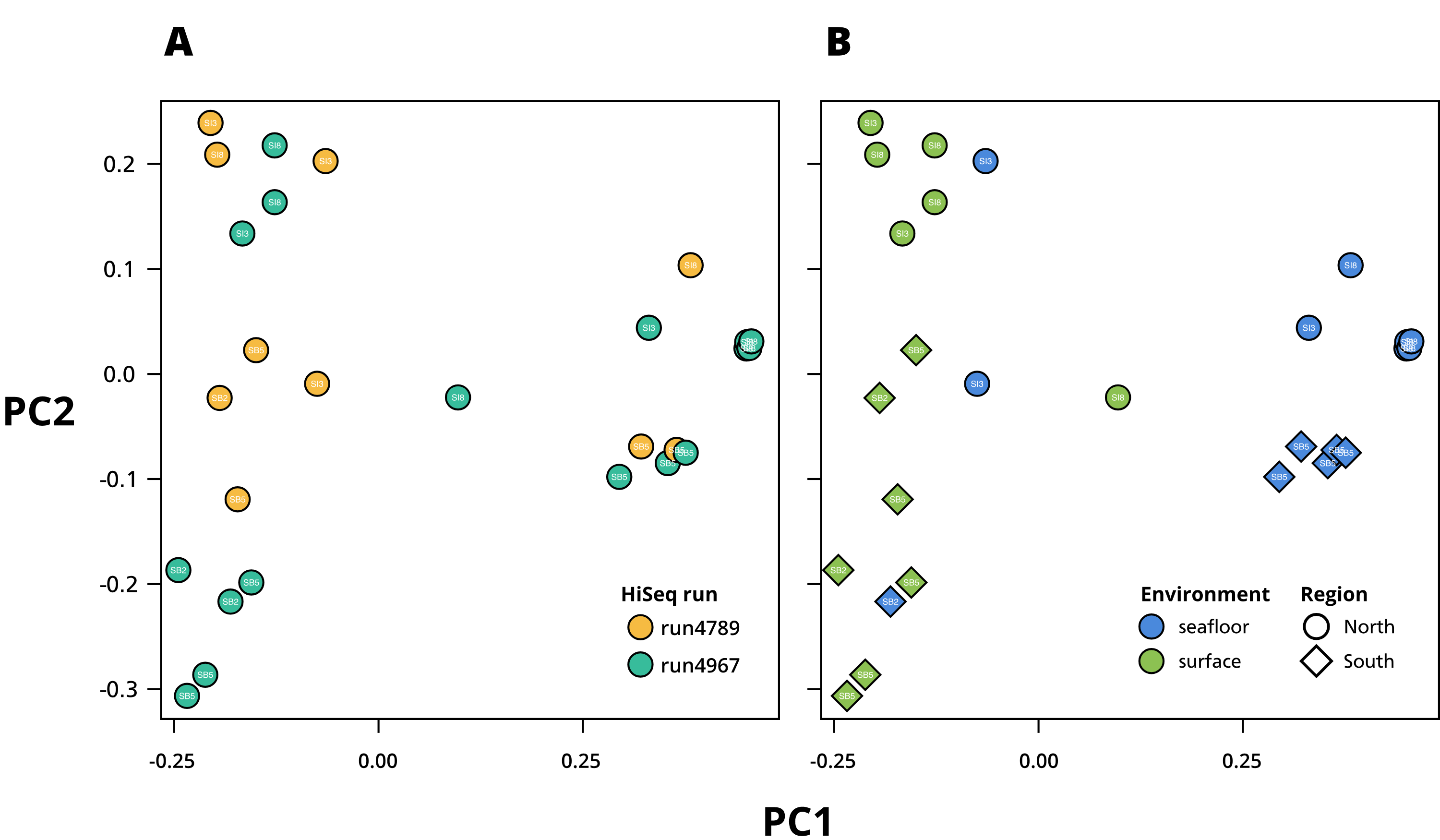

Supplement: Supplemental Information 2 — (A) Experimental variable. (B) Environmental and geographic variables. [file peerj-09-11112-s002.png]
